# Clinical profile and treatment outcome of patients with ileo-sigmoid knotting, an experience from Ethiopian setting: a six years review

**DOI:** 10.1186/s12893-025-02859-z

**Published:** 2025-04-22

**Authors:** Dawit Alemayehu Bitewa, Tirufat Dagmawi, Mandante Bogale, Mezgebu Miskir, Dagmawi Abiy, Megbar Dessalegn

**Affiliations:** 1 Department of Surgery, School of Medicine, Debre Markos University, Debre Markos, Ethiopia; 2 Department of Gynecology and Obstetrics, School of Medicine, Debre Markos University, Debre Markos, Ethiopia; 3 Department of Pathology, School of Medicine, Debre Markos University, Debre Markos, Ethiopia

**Keywords:** Ileo-sigmoid knotting, Intestinal obstruction, Surgical outcomes

## Abstract

**Background:**

Ileo-sigmoid knotting (ISK) is a rare cause of intestinal obstruction, characterized by the twisting of the ileum around the sigmoid colon or vice versa. This study aimed to assess the clinical characteristics and treatment outcomes of patients undergoing laparotomy for ISK at a tertiary hospital in Ethiopia.

**Methods:**

This is an institution based cross sectional study conducted at Debre Markos Comprehensive Specialized Hospital in Debre Markos City, Northwest Ethiopia. A six-year study was conducted at Debre Markos Comprehensive Specialized Hospital by revewing the medical records of 42 patients operated for ISK between March 31, 2018, and April 1, 2024. Data were extracted, processed, and analyzed using Epi-Data 4.6 and STATA 17.0. Fisher’s exact test was used to determine statistical significance (p-value ≤ 0.05).

**Results:**

Thirty-eight patients (90.5%) had complete medical records, with a mean age of 39.2 years (SD ± 10.2) and a male predominance (M: F = 3.2:1). Accurate preoperative diagnosis was achieved in only 5.3% of cases, with the majority being misdiagnosed as small or large bowel obstruction. Gangrene of both the ileum and sigmoid colon was observed in 71.1% of cases. The most commonly performed procedure (68.4%) was resection of both segments with primary ileo-ileal and colorectal anastomosis. Postoperative complications occurred in 52.6% of patients, the most common one being anemia (31.6%). Mortality was 7.9% and was significantly associated with anastomotic leaks (*p* = 0.045). The average hospital stay was 8.2 days (IQR: 6–37).

**Conclusions and recommendation:**

The accuracy of preoperative diagnosis of ileo-sigmoid knotting in this study is lower. However, ileo-sigmoid knotting had high postoperative morbidity and mortality. This study highlights the need for heightened awareness for preoperative diagnosis and prompt surgical treatment. We recommend a prospective multicentric study to guide on appropriate operative decision making in ISK patients.

## Introduction

Ileo-sigmoid knotting (ISK) is a rare cause of intestinal obstruction resulting from the wrapping of the ileum around the sigmoid colon and its mesentery or vice-versa. if left untreated, it can rapidly progress to gangrene of the ileum and/or the sigmoid colon [1, 2]. It generally occurs in areas where the incidence of sigmoid volvulus (SV) is higher commonly referred to as the volvulus belt [3–5]. The incidence of ISK is not well known. It accounts for 18–50% of cases of SV in eastern countries and 5–8% in western countries [3]. The development of ISK is reliant on three factors: a long small bowel mesentery with free mobile small bowel [6, 7], a long sigmoid with a narrow pedicle [6, 8], and a bulky diet with the presence of an empty small bowel [6–8].

Preoperative diagnosis is difficult to ascertain due to its rarity and atypical radiographic findings [9, 10], making it essential to differentiate it from sigmoid volvulus as attempts to deflate the distended colon with sigmoidoscopy or a rectal tube may be dangerous [8, 11]. Use of contrast-enhanced computed tomography (CT) appears sensitive tool for the diagnosis of ileo-sigmoid knotting with diagnostic accuracy of more than 90% [12–14]. However, this may not be accessible in most settings where sigmoid volvulus is prevalent [6, 8, 15]. Furthermore, a delay in intervention results in rapid progression and gangrenous change with fatal complications related to generalized peritonitis, sepsis, dehydration, and/or electrolyte imbalance [4, 8, 13].

Accordingly, early diagnosis and expedited emergency laparotomy is paramount. The decision on the type of procedure to be performed depends on the bowel viability and patients’ general condition. This could range from a simple detorsion to resection of significant portion of the bowel with or without colostomy [8, 16]. There are few cases of ileo-sigmoid knotting described in the literature and conclusions are drawn from smaller sample [1, 7]. Hence this study aims to shed light on the clinical profile, intraoperative findings, and challenges in the preoperative diagnosis and management and treatment outcomes of ISK in a resource-limited setting. The study will be able to provide data to guide clinicians in choosing appropriate surgical interventions based on intraoperative findings evaluating the applicability. The study will serve as an input for larger multicenter studies aimed at standardizing care for ISK and contribute to the development of guidelines or algorithms for the management of ISK.

## Methods

### Study setting, design and period

This is an institution based cross sectional study conducted at Debre Markos Comprehensive Specialized Hospital (DMCSH) in Debre Markos City, Northwest Ethiopia. DMCSH is a teaching hospital with 300 beds that serves a catchment population of over five million people. The study reviewed procedures performed on patients with a diagnosis of ileo-sigmoid knotting from March 31, 2018 to April 01, 2024 over a period of six years.

### Source population and study population

The source population comprised all patients who underwent laparotomy for suspected Intestinal Obstruction at Debre Markos Comprehensive Specialized Hospital. The study population specifically included those who received a confirmed diagnosis of ileo-sigmoid knotting following laparotomy during the study period.

### Eligibility criteria

#### Inclusion criteria

Patients were eligible for inclusion if they had a confirmed diagnosis of ileo-sigmoid knotting established during laparatomy.

#### Exclusion criteria

Those with incomplete charts, defined as those without at least one progress note or discharge summary, Patients who were transferred from another hospital after laparotomy were excluded from the study (because it is difficult to retrieve full pre-operative data).

### Variables of the study

#### Dependent variable

The treatment outcomes of ileo-sigmoid knotting (postoperative morbidity and mortality).

#### Independent variables

- ✓ Socio-demographic characteristics: Age, sex, residence, mode of arrival, and referral status.
- ✓ Clinical presentation: Hypotension, tachycardia, fever, duration of symptoms, sepsis, comorbidity, use of prophylactic antibiotics, previous surgery, vasopressor use, blood transfusion.
- ✓ Preoperative investigations: leukocytosis, hematocrit, and imaging studies.
- ✓ Intraoperative findings: duration of anesthesia, duration of surgery, blood transfusion, vasopressor use, bowel ischemia, peritoneal contamination, and type of surgical procedure performed.
- ✓ Post-operative variables: intensive care unit admission, re-laparotomy, and immediate post-operative shock.

### Data collection tool and method

Data were collected by three general practitioners using a data extraction checklist prepared based on reviewed literature. To ensure reliability, the tool was evaluated by two general surgeons, and a pretest (5%) was conducted before the actual data collection to check its relevance for the extraction of appropriate data. The data were cleaned, coded, entered into Epi-data version 4.6, and exported to STATA version 17.0 for analysis. The results were presented as mean, proportions or frequency in text or tables. Fisher’s exact test was employed to assess the significance of differences in treatment outcomes, at a p-value ≤ 0.05.

## Results

### Socio-demographic characteristics of the patients

A total of 38 patients (90.5% with complete charts) who underwent emergency laparotomy for ileo-sigmoid knotting were included in the study. The majorities were males (M: F 3.2:1). The mean age of the patients was 39.2 years (SD ± 10.2). The majority (30, 78.9%), were referred from other health institutions (Table 1).

**Table 1:** Sociodemographic characteristics of the patients, *DMCSH*,* Mar 2018 – Apr 2024*

Variable		Frequency	Percent
Age (years)	< 20	1	2.6
	20–29	3	7.8
	30–39	15	39.4
	40–49	11	28.9
	50–59	6	15.8
	>=60	2	5.2
Sex	Male	29	76.3
	Female	9	23.7
Residence	Rural	31	81.6
	Urban	7	18.4

### Clinical presentation of the study participants

The majority of (30, 78.9%) patients presented within the first day of symptom onset and the mean duration of symptoms was 1.39 (SD ± 0.85) days. Two patients had a history of previous surgery, while another two patients (5.3%) were in their third trimester of pregnancy. Only one patient had comorbidity which is type-2 diabetes mellitus.

More than three-fourths (29, 76.3%) were tachycardic and 8(21.1%) were in shock at presentation. In laboratory studies, 26 (68.2%) had leukocytosis. Twenty two patients had plain abdominal x rays; of these 14(63.6%) had features of small bowel obstruction while features of large bowel obstruction and viscous perforation was suggested in 7(31.8%) and one (4.6%) patient respectively.

**Table 2:** Clinical profile of the patients, DMCSH, Mar 2018 – Apr 2024

Symptoms	Frequency	Percent
Abdominal pain	38	100
Vomiting	36	94.7
Failure to pass faces and flatus	35	92.1
**Signs**		
Shock at presentation	8	21
Abdominal tenderness	29	76.3
Guarding	13	34
Hyperactive bowel sound	9	25
Hypoactive bowel sound	19	52.8
Hyper-tympanic to percussion	29	80.6
Stool in the rectum	18	47.4
Blood in the rectum	2	5.26

### Preoperative diagnosis of the study participants

A documented accurate preoperative diagnosis of ileo-sigmoid knotting was made in only two (5.3%) out of thirty-eight patients and the majority (21, 55.3%) were diagnosed with small bowel obstruction; whereas the remaining 13(34.2%) with large bowel obstruction and two had perforated viscous. There was no rectal tube deflation trial.

### Intraoperative findings and types of procedure performed

The ileum was an active component of the obstruction loop in 35(91.9%) patients, whereas the sigmoid colon was an active component in remaining 3 (8.1%) patients. Both ileum and sigmoid colon were found to be gangrenous in the majority (27, 71.1%), of the patients. The most common procedure (26, 68.4%) performed was resection of both segments with primary ileo-ileal and colorectal anastomosis, whereas 7 (18.4%) patients underwent resection of both segments with ileo-ileal anastomosis and Hartmann’s procedure (Table 3). The mean duration of the procedure was 120.9 (SD ± 39.6) minutes.

**Table 3:** Intraoperative findings and types of procedures performed for the patients. DMCSH, Mar 2018 – Apr 2024

Intraoperative finding	Type of procedure performed	Frequency	Percent
Both gangrenous	Primary ileo-ileal and colorectal anastomosis	19	50
	Primary ileo-ileal anastomosis, with Hartman’s procedure	7	18
	Ileostomy and Hartman’s procedure	1	2.6
Gangrenous ileum and viable sigmoid colon	Primary ileo-ileal anastomosis with sigmoid resection colorectal anastomosis	7	18.4
	Primary ileo-transverse anastomosis and sigmoid de-rotation	1	2.6
Viable ileum and gangrenous colon	Sigmoid resection and colorectal anastomosis	2	5.26
Both viable	Sigmoid resection and colorectal anastomosis	1	2.6

Five patients (13.6%) experienced hemodynamic instability intraoperatively and required inotropes. After the procedure, 33 (86.8%) patients were transferred to post anesthesia recovery unit, whereas the remaining 5 (13.6%) patients were admitted to the intensive care unit.

Among the 38 patients 20(52.6%) developed one or more complications postoperatively. The most common postoperative complications were anemia and hospital acquired pneumonia, which occurred in 12 (31.6%) and 9(23.9%) of all the patients respectively. Among the eight (21.1%) patients who developed surgical site infections, four required re-laparotomy. The other three (10.53%) patients who required relaparotomy were among the patients who developed anastomotic leaks, accounting for 6 (15.8%) of all the patients (Table 4).

**Table 4:** Postoperative complications; and factors associated with mortality in the patients, DMCSH, Mar 2018 – Apr 2024

Type of complication	Number of patients	Percent*
Anemia	12	31.6
Hospital acquired pneumonia	9	23.9
Surgical site infection	8	21.1
Anastomotic leak	6	15.8
Hypokalemia	3	7.9
Wound dehiscence	3	7.9
Acute renal failure	2	5.3

Three patients died postoperatively. All patients had gangrenous ileum and sigmoid colon and died after re-laparotomy. Re-laparotomy was significantly associated with increased mortality (*p* = 0.002) (Table 5). Additionally, mortality was significantly associated with anastomotic leaks (*p* = 0.045). Patients with anastomotic leaks had a higher mortality rate compared to those without leaks. In addition, the presence of guarding and rigidity was found to be significantly associated with the occurrence of one or more complications (*p* = 0.006). This indicates that guarding and rigidity may play a role in predicting the occurrence of complications. In contrast, shock at presentation, pre-operative anemia, leukocytosis, and the finding of blood on the examining finger during digital rectal examination were not significantly associated with postoperative complications or mortality. The median hospital stay was 8.2 days with an inter quartile range of 6–37 days.

**Table 5:** Factors associated with mortality in the patients, DMCSH, Mar 2018 – Apr 2024

Variables	Category	Mortality		
		Yes	No	P-value
Sex	M	2	27	0.567
	F	1	8	
Age (years)	< 60	3	33	0.897
	> 60	0	2	
Residence	Rural	1	5	0.081
	Urban	2	30	
Duration of symptoms	< 24 h.	2	28	0.119
	>=24 h.	1	7	
Gangrenous bowel part	Double segment	3	27	0.063
	Single segment	0	7	
Shock on presentation	Yes	2	6	0.106
	No	1	29	
Anastomotic leak	Yes	2	4	0.045
	No	1	31	
Intraoperative shock	Yes	1	4	0.253
	No	2	31	
Re-laparotomy	Yes	3	3	0.002
	No	0	32	

## Discussion

The management of ileo-sigmoid knotting (ISK) presents significant challenges, particularly in resource-limited settings where timely diagnosis and effective treatment are often hindered. This study explores the clinical profile, intraoperative findings, and the obstacles faced in the preoperative diagnosis and management of ISK. By examining treatment outcomes, the study aims to provide valuable insights that can assist clinicians in selecting appropriate surgical interventions based on intraoperative observations. Additionally, the findings are intended to inform larger multicenter studies and contribute to the development of standardized guidelines or algorithms for the effective management of ileo-sigmoid knotting.

Among the participants analyzed, patients diagnosed with ISK were relatively younger, with an average age of 39.2 ± 10.2 years. This finding is consistent with the results reported in previous studies [1, 7, 8, 17–19]. Male patients were 3.22 times more affected than female patients, a trend that aligns with earlier findings [7, 8, 18, 19]. The patients in this study sought medical attention after a shorter duration of symptoms, averaging 1.39 days, compared to findings reported in other studies [7, 8, 20]. However, despite early presentation, patients experienced a relatively higher rate of postoperative complications [6, 8]. Two studies showed no correlation between the duration of symptoms and gangrene frequency, which was also observed here [6, 11].

In 92.1% of cases, the obstruction involved the ileum, while gangrene was observed in both the ileum and sigmoid colon in 71.1% of patients [6, 7]. Preoperative diagnosis of ISK remains difficult, with diagnostic accuracy below 25% due to nonspecific clinical and imaging findings [6, 7]. A recent case report from Ethiopia highlighted this challenge, emphasizing that ISK is often not suspected preoperatively, leading to delays in surgical intervention [21]. This study supports previous research indicating that features of compound obstruction, such as dilated large and small bowel loops with multiple air-fluid levels, should raise suspicion for ISK [1, 6–8, 20]. CT scans may show medial deviation and a pointed appearance of the distal descending colon, along with a radial pattern of mesenteric vasculature. While contrast-enhanced CT is highly accurate for diagnosing ISK, its limited availability in low-resource settings necessitates the optimization of alternative diagnostic approaches. Bedside ultrasound and plain radiography, though less specific, can serve as crucial tools for early detection. Future guidelines should include training for clinicians in low-resource settings to improve the interpretation of these imaging modalities. Unlike sigmoid volvulus, the “swirl” sign in ISK appears in more consecutive CT slices. Since ISK requires emergency surgical intervention, distinguishing it from sigmoid volvulus is critical. However, resource limitations and patients’ hemodynamic stability often restrict the use of advanced imaging, making urgent laparotomy essential, this issue is more pronounced in low resource setting like the one this study is conducted, where none of the patients have had a CT scan [6, 17, 22].

Surgical intervention should be guided by the patient’s physiological status, operative risks, and intraoperative findings [1, 6–8, 17, 20, 22–25]. Traditionally, resection and primary anastomosis of the ileum with colostomy for the sigmoid colon has been recommended for managing gangrenous segments. Of the 27 patients (71.1%) with gangrenous ileum and sigmoid colon, 19 (70.4%) underwent successful primary resection and anastomosis, aligning with newer studies supporting this approach [8, 17, 18, 22].

In gangrenous cases, the sigmoid colon is resected, and a primary anastomosis is performed if the patient is stable and a tension-free connection is possible. Gangrenous small bowel segments are also excised, with continuity restored via entero-enterostomy. In stable, non-gangrenous cases, careful knot untying may suffice, though volvulus-preventive procedures like mesopexy, mesoplasty, or resection with primary anastomosis may be added [6].

Primary small bowel anastomosis is preferred, but end-to-end anastomosis should be avoided if the terminal ileum is gangrenous within 10 cm of the ileocecal valve. In such cases, an end-to-side ileo-cecostomy is recommended after closing the distal stump. Sigmoid colon resection is often advised even when viable due to the risk of recurrent volvulus or knotting. Many authors recommend a Hartmann’s procedure or covering colostomy in emergencies to prevent fecal leaks, though recent trends favor primary colonic anastomosis when the bowel is clean, well-vascularized, and non-distended. Intraoperative colonic irrigation followed by resection and primary anastomosis is another option [6, 26]. Although resection and anastomosis were successful in 70.4% of gangrenous sigmoid cases, 21.1% of patients who underwent double anastomosis (ileum and sigmoid) developed an anastomotic leak. This underscores the need for clear guidelines or algorithms to guide patient management and minimize complications.

Of the 38 patients in our study, 52.6% developed one or more postoperative complications, a rate consistent with findings in some studies, which reported complication rates ranging from 40 to 65% [1, 4, 6]. The most common complications in our cohort were anemia and hospital-acquired infections, occurring in 31.6% and 23.9% of patients, respectively. Anemia rates were comparable to those reported in other studies, while hospital-acquired infections were higher than some studies reporting 15% but lower than others reporting up to 35% [9, 10, 18]. Surgical site infections occurred in 21.1% of patients, a rate consistent with findings of 20–25% in some reports, though others have reported rates as low as 10% [20, 27]. Anastomotic leaks accounted for 15.8% of cases, aligning with rates documented in some studies, which ranged from 8 to 20% [4, 6, 14]. Mortality was recorded in 7.9% of patients, which is within the range reported in other studies, varying from 5 to 20% [4, 9, 17]. Additionally, mortality was significantly associated with anastomotic leaks (*p* = 0.045), consistent with findings in the literature, though some studies reported a weaker correlation [6, 10, 14].

## Conclusion and recommendation

The pre-operative diagnosis accuracy for ileo-sigmoid knotting in this study was low, reflecting nonspecific clinical and imaging finding associated with the condition. The post-operative complications were relatively higher in this study despite early presentation to the healthcare, this could be potentially due to high prevalence of gangrenous bowel. The findings highlight the importance of developing standardized guidelines or algorithms to help in pre-operative diagnosis and in deciding surgical methods. Particularly the high rates of anastomotic leaks in patients undergoing anastomosis highlights the need for careful patient selection and tailored surgical approaches limit the risk. To improve patient outcomes, we recommend the development of standardized clinical guidelines, including: Risk stratification criteria based on patient demographics and intraoperative findings: Early imaging protocols to enhance preoperative diagnostic accuracy; Operative decision-making pathways to optimize the choice of surgical procedures and reduce postoperative complications; Postoperative monitoring protocols with specific thresholds for early detection of anastomotic leaks and other complications. Future researches should focus on multi centered studies with larger sample sizes to better evaluate risk factors associated with post-operative complications and mortality. Those studies could provide a better understanding of the clinical cores and optimal management strategies.

### Ethical approval and consent to participate

Ethical approval for the study was obtained from the Institutional Review Board of Debre Markos University College of medicine and health sciences. The need for written informed consent was waived by the ethical review board and permission to access patient records was granted by Debre Markos comprehensive specialized Hospital. Patient confidentiality was maintained by using anonymous data extraction forms and securely storing the data.

### Limitations of the study

This single-center retrospective study may limit generalizability and introduce selection bias due to incomplete documentation; the absence of data on surgeon experience prevents assessment of its impact on outcomes. Additionally, the small sample size reduces statistical power, particularly in subgroup analyses like anastomotic leaks. Reliance on medical records may have omitted key clinical details. Future multicenter prospective studies with larger samples are recommended for more comprehensive insights.

## Data Availability

The raw data can be accessed in response to reasonable requests from the corresponding author.
